# Pregnancy Ketonemia and Development of the Fetal Central Nervous System

**DOI:** 10.1155/2018/1242901

**Published:** 2018-06-04

**Authors:** Agata Bronisz, Mateusz Ozorowski, Magdalena Hagner-Derengowska

**Affiliations:** ^1^Endocrinology and Diabetology, Faculty of Medicine, Ludwik Rydygier Collegium Medicum in Bydgoszcz, Nicolaus Copernicus University in Toruń, 9 Skłodowskiej-Curie Street, 85-094 Bydgoszcz, Poland; ^2^Clinical Neuropsychology, Faculty of Health Sciences, Ludwik Rydygier Collegium Medicum in Bydgoszcz, Nicolaus Copernicus University in Toruń, 9 Skłodowskiej-Curie Street, 85-094 Bydgoszcz, Poland

## Abstract

Glucose is the major source of energy for the human brain which in turn uses ketone bodies as a supplement for energy deficit in glucose cell deficiency conditions. Pregnancy complicated by gestational diabetes is a condition associated with significantly increased risk of ketonemia development. The data available proves a changing influence of ketones on the central nervous system during fetal life and in adults as well. Ketone bodies freely pass through the placenta. They can affect fetal growth and organ damage development, especially the central nervous system. As agreed in the current recommendation of the diabetes associations, it is not obligatory for the attending doctor to conduct a routine inspection of ketone bodies during diabetes treatment in pregnancy. This article is a literature review of ketones' effect on the central nervous system and an attempt to initiate discussion whether we should consider including ketonemia assessment into the standard care package for pregnant women with diabetes and begin some research on the explanation of its influence on fetal development.

## 1. Ketogenesis

*β*-Hydroxybutyrate, acetoacetate, and acetone ketones are intermediate metabolites of oxidation, mainly of fatty acids. Some amino acids called ketogenic amino acids can act as another starting material for the production of the abovementioned. Phenylalanine, isoleucine, leucine, lysine, tryptophan, and tyrosine can be listed among them [1]. Ketone synthesis takes place mainly in the liver, in a mitochondrial matrix of hepatocytes, and is regulated hormonally. Hormones stimulating lipolysis (glucagon, epinephrine, norepinephrine, adrenocorticotropic hormone, thyroid-stimulating hormone and growth hormone, vasopressin, estradiol, and testosterone) which cause increased blood concentration of free fatty acids (FFA)—the starting materials for ketone body production—enhance ketone formation. Glucagon is the first hormone stimulating FFA release from adipose tissue, and its molar ratio to insulin increases at the beginning of starvation [2]. Insulin, on the other hand, inhibits lipolysis and reduces ketone synthesis [3–5].

**Figure 1:**
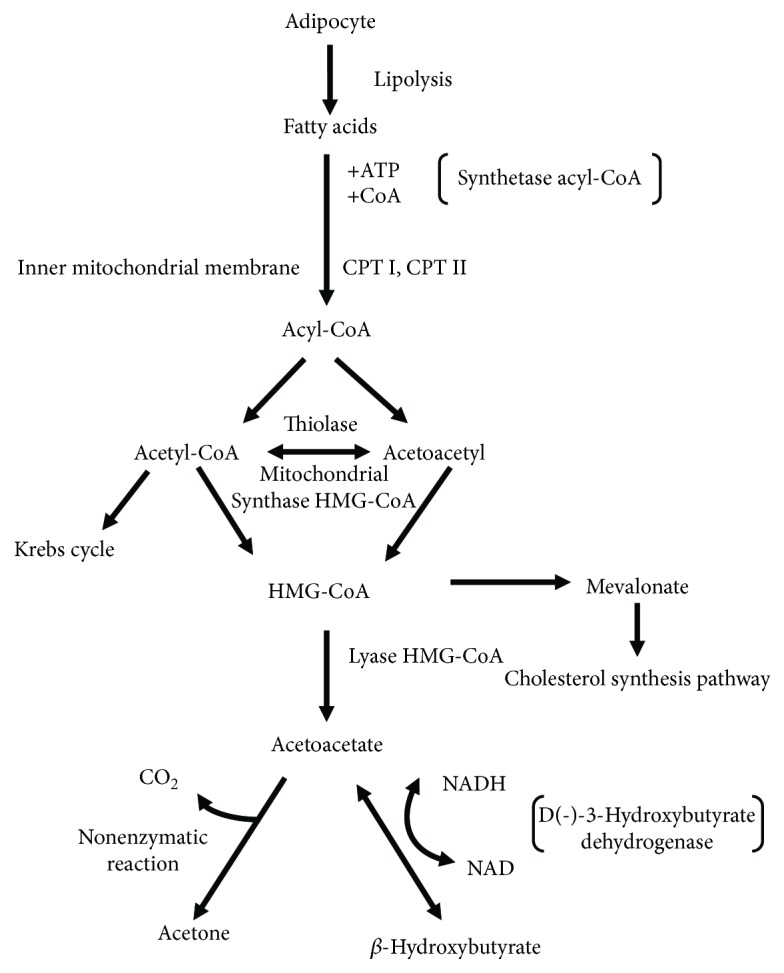
Ketogenesis scheme.

FFA released from fat tissue along the blood vessels reach the liver and pass into the hepatocyte cytoplasm where in turn they are conjugated with coenzyme A, which results in the formation of acyl-coenzyme A (acyl-CoA) that passes into the mitochondrial matrix with the participation of carnitine transporter. Transport intensification is triggered by the influence of the activation of nuclear peroxisome proliferator-activated receptors (PPAR). The activation of their alpha isoforms (PPARa) causes among others the increase of the expression of genes encoding palmitoyltransferases I and II, which are enzymes responsible for the transport of amino acyl-CoA from cytoplasm to mitochondrion [4, 6–8]. In mitochondria, in the process of *β*-oxidation, acyl-CoA is oxidized into several molecules of acetyl-CoA that may be used in the Krebs cycle. In order to access the Krebs cycle, the acetyl-CoA needs the presence of oxaloacetate, whose concentration decreases when carbohydrates are not available. The excess of acetyl-CoA that has not been metabolized in the Krebs cycle cannot be used for feedback glucose production [2]. It is metabolized sequentially into 3-hydroxy-3-methylglutaryl-CoA and then into acetoacetate (Figure 1) [9].

In mitochondrion, the acetoacetate is converted to *β*-hydroxybutyrate under the influence of D(-)-3-hydroxybutyrate dehydrogenase which is dependent on nicotinamide adenine dinucleotide (NAD). The ratio of both compounds is equal under physiological conditions, and the before mentioned enzyme converts the two compounds into each other while depending on the oxyreduction balance inside mitochondrion [1]. In glucose deficiency states, when a reduced form of nicotinamide adenine dinucleotide (NADH) dominates, there is a shift of the reaction towards *β*-hydroxybutyrate formation [9–11]. In these states, the ratio of *β*-hydroxybutyrate to acetoacetate in the blood ranges from 3 : 1 to 10 : 1. Furthermore, acetoacetate is converted into acetone as a consequence of a spontaneous (nonenzymatic) and irreversible reaction [12]. Ketones, which act as acidic compounds, bind blood bicarbonates and contribute to lowering serum pH [2].

### 1.1. Ketone Bodies as an Alternative Energy Source

**Figure 2:**
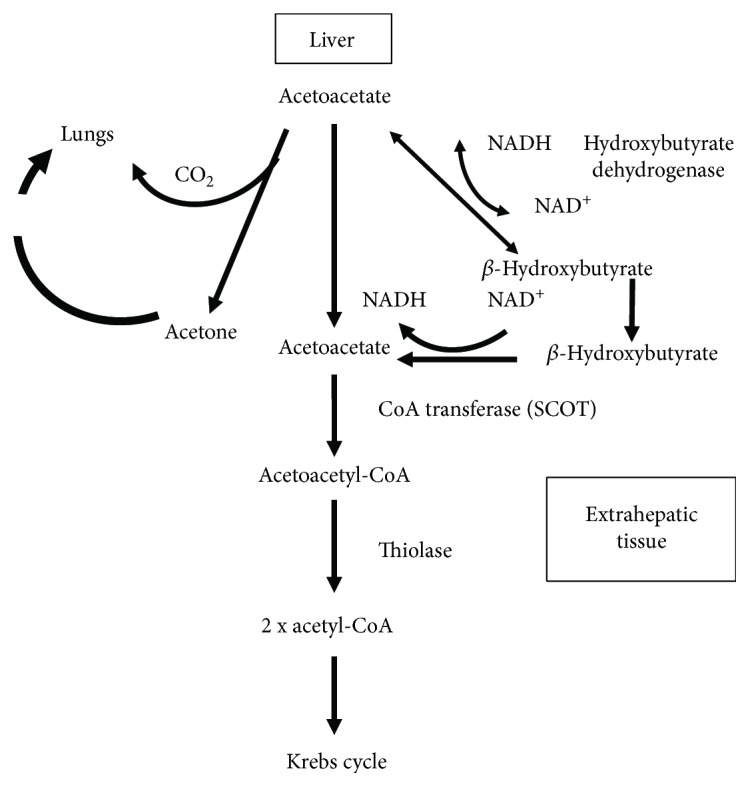
Tissue utilization of ketone bodies.

FFA become the main substrate of the body energy in the state described as intracellular glucose deficiency. Ketone bodies, which are formed from the FFA, become the main source of energy for organs and tissues. Acetoacetate and *β*-hydroxybutyrate as substances that are soluble in aqueous solutions are easily transported from the liver to peripheral tissues as they require no albumin or lipoprotein for this process [2]. Ketones pass into neurons and glial cells through the conveyor monocarboxylic acids (MCT (monocarboxylic acid transporters)). The basic isoform for this process is isoform 1, localized in astrocytes, oligodendrocytes, and endothelial cells [13]. In the target cells, the acetoacetate is converted back into two molecules of acetyl-CoA that are included in the Krebs cycle (Figure 2). The enzyme responsible for this process—ketoacyl-coenzyme transferase—is not present in liver mitochondria; therefore, ketones cannot be the source of energy in hepatocytes [2]. *β*-Hydroxybutyrate, on the other hand, acts more as a “reserve power” that requires its transformation into acetoacetate with the participation of *β*-hydroxybutyrate dehydrogenase described above [1, 12].

Increased ketogenesis in physiological conditions is observed not only in the fasting state but also during an energy-balanced diet that includes high fats and a low-carbohydrate level, as well as after exercise, during pregnancy and in the neonatal period. In pathological conditions, increased synthesis of ketone bodies frequently occurs in alcoholic disease and uncontrolled diabetes [12, 14–16]. Ketones are metabolized in important vital organs such as the brain, heart, and kidneys. They are energy material also for skeletal muscle. Yet, as described above, the liver lacks the ability of using them as the source of energy [1, 17].

Ketones take an important part in the central nervous system (CNS) where they become the sole source of energy in the intracellular glucose deficiency states owing to the fact that they go freely through the blood/brain barrier, whereas FFA do not [2]. In the animal model, Blázquez et al. proved that ketones in the CNS come not only from the liver synthesis but also astrocytes that regulate glucose metabolism in the brain are capable of producing ketones [18]. *β*-Hydroxybutyrate and acetoacetate that have been mentioned above are energy substrate cells. Stenerson et al. proved that *β*-hydroxybutyrate and lactate accumulate in the brain basal ganglia among children with ketoacidosis [19]. A limited use of acetone through the cell results from its physical properties. Acetone is removed from the exhaled air and urine due to its high volatility [1, 11, 20].

### 1.2. Ketone Body Metabolism among Pregnant Women

Many metabolism modifications occur in a woman's body during pregnancy. One of the reasons for their occurrence is the appropriate use of energy resources. Body fat changes are of particular importance in these processes. In early pregnancy, the mother's body accumulates adipose tissue supply. As a result of increased production of placental lactogen, progesterone, and prolactin, the appetite of a pregnant woman is strongly stimulated. Acceleration of insulin being produced is also observed. Hyperinsulinemia increases lipogenesis and decreases lipolysis, which results in accumulation of fat reserves. In the second half of pregnancy, under the influence of increasing placental hormones and cytokine concentration, catabolic processes can be observed [21–23]. Lipolysis increases availability and use of FFA as the energy material for the mother's body in place of glucose that is consumed mostly by the fetus. These mechanisms are responsible for increased ketogenesis during pregnancy and are three times higher at night among pregnant women than among nonpregnant women [11, 14, 22, 24, 25]. There are observations documenting the development of ketoacidosis among healthy pregnant women even during starvation [11, 16, 26]. An extraordinary predisposition to the development of ketonemia may be developed among pregnant women with glucose intolerance. In these patients, typical pregnancy metabolic processes overlap with recommendations to restrict carbohydrates consumption in a diabetic diet, which creates favorable conditions for increased ketogenesis. Ketonuria is commonly diagnosed when tested after fasting and often correlates with increased levels of *β*-hydroxybutyrate in the blood [27].

Ketones pass freely across the placenta and can be used as a source of energy by the fetus too [1, 28]. Seccombe et al. served pregnant rats a radioisotope-labelled *β*-hydroxybutyrate through the femoral vein and proved that this isotope had been detected in fetal plasma already after five minutes. Carbons derived from a labelled *β*-hydroxybutyrate were present in acyloglycerols, phospholipids, and cholesterol [29].

## 2. Ketones and the Central Nervous System

The observational and experimental studies on animals revealed that ketone bodies in the CNS not only are a source of energy but also have an impact on its functioning. Yet, the range and mechanism of ketones' influence on the CNS has not been completely understood yet. The impact of ketonemia on the CNS excitability has been documented. The first clinical descriptions of the use of keto diet as an effective seizure treatment are dated back to the beginning of the twentieth century. This method, withdrawn after the introduction of diphenylhydantoin to epilepsy treatment, was used again as an alternative way in cases of drug-resistant seizures in the late 90s. Its efficacy does not correlate with ketone level in serum [30]. Some reports on beneficial effects of ketone bodies in Huntington's disease are also to be found [7].

There are several theories explaining the mechanisms of ketones' impact on the CNS functioning. According to one of them, ketone metabolism causes increased synthesis of andenozyno-5′-triphosphate (ATP) in mitochondria. Increased energy reserves in the cells result in a reduction of ATP production via glycolysis. Glycolysis enzymes are associated with membrane proteins. It leads to lowering ATP compartments located in cell membrane. This situation may reduce ATP-dependent pumps' activity in membrane channels, which can contribute to the stabilization of cell membranes and affect the neuron activity [31].

According to another theory, acetoacetate directly inhibits vascular glutamate transporter's activity in hippocampal synaptic vesicles that are responsible for synaptic transport of this important neurotransmitter. The acetoacetate does not have an inhibitory effect on different neurotransmitter's transport—gamma aminobutyric acid (GABA). It has been shown, however, that the keto diet inhibits the activity of substantia nigra pars reticulata, where the main GABAergic neurons are located. According to another hypothesis, ketones stimulate GABA synthesis that is the inhibitory neurotransmitter of brain excitability [31]. It has been reported that the *β*-hydroxybutyrate does not show inhibitory actions described.

Another theory explains the inhibition of substantia nigra by a G protein of ATP-dependent membrane channel that is associated with the GABA receptors or A1 adenosine receptors. Adenosine decreases neuron activity by means of A1 receptor. The ketogenic diet causes increased levels of adenosine that disappear after the consumption of carbohydrates. An idea, according to which the lack of glucose may have more importance for neurocyte functioning than ketone activity, has also been taken into consideration [31].

Last years' observations bring some information about beneficial effects of ketone bodies on the CNS functioning in two most common degenerative diseases: Parkinson's disease and Alzheimer's disease [7]. In this area, *β*-hydroxybutyrate, which is a histone deacetylase inhibitor, a key enzyme involved in the epigenetic regulation of gene expression, has proved to be very active. This way, a ketone may influence many metabolic processes related to aging and cells' death. It has been shown that *β*-hydroxybutyrate (i) reduces neuronal apoptosis, including the one associated with hypoglycemia; (ii) increases the number of motor neurons; (iii) increases their activity and vascularization; and (iv) protects neuronal cultures against developing the amyloid pathology [32, 33]. Moreover, it has been suggested that ketones may be of great importance because of increasing the concentration of calbindin—an intracellular calcium-binding protein that prevents the reduction of the dopamine concentration in the cells, and it can lower the Parkinson's disease symptoms with this mechanism [32]. Also, in a traumatic brain injury, some beneficial effects of ketone bodies have been observed, for example, ketones have reduced the area of damage and brain edema. Infusions of *β*-hydroxybutyrate have not only improved cognitive and sensory motor functions but they have also influenced learning and memorizing processes favorably [7]. Decreasing anxiety and depression has also been noted. The abovementioned effect is associated with reduction of hippocampal neuronal loss [32].

Glaser et al. compared the effects of hyperglycemia and ketonemia on the functions of the six cortex areas and two striatum nuclei areas in rats. The authors evaluated three parameters in both states: cerebral water distribution, cerebral blood flow, and content of selected cellular metabolites including high-energy compounds by magnetic resonance spectroscopy. A significantly decreased cerebral blood flow, altered water distribution in the cerebral cortex and in ketosis, and reduced content of high-energy compounds were found in both: hyperglycemia and ketonemia. The authors also suggest the possible influence of insulin deficiency and low levels of IGF-1 excluding ketones themselves on the reduction of cerebral blood flow [34].

### 2.1. Ketones and the Fetal Central Nervous System

The literature on the influence of pregnancy ketonemia on fetal development is limited and in contradiction with the beneficial effects of ketone bodies on the CNS described in adults. Kurepa et al. suggest that the mother's hyperketonemia can take part in fetal development during pregnancy [35]. According to Rizzo et al., the *β*-hydroxybutyrate concentration correlates with neuropsychological offspring's development in the third trimester of pregnancy [36]. These observations support the research of Sussman et al. who analyzed the impact of normocaloric ketogenic diet on CD-1 mice progeny's development. The diet was being applied to animals for 30 days before pregnancy, and it was continued during pregnancy and lactation. Development of the fetuses and their internal organs was assessed with magnetic resonance method in two periods of pregnancy. It was found that in the first half of pregnancy, the fetuses on a ketogenic diet had a significantly higher growth, which was significantly inhibited later on. Finally, the offspring of ketogenic mothers showed total embryo growth abnormalities and reduced volume and distortion of the internal organs. Studies have shown reduction of the cerebral cortex volume, hippocampus, corpus callosum, lateral brain ventricles, larynx, and thymus [37]. There was, however, a larger volume of the cerebellum, spinal cord, left ventricular heart, and skeletal muscle [32]. The data on hypothalamus is inconclusive in another observation of the same team: in the above-cited study, the hypothalamus was found to have enlarged, and in the second one, its volume was reduced in mouse fetuses' ketogenic mothers [32, 37]. According to the authors, decrease of placental transfer ketones and increased expression of the glucose transporter GLUT1 in this period explain the dominant inhibitory effect of ketogenic diet on fetus growth in the second half of pregnancy, which, in their opinion, also indicates that glucose is essential for intensive fetus growth in late pregnancy [37].

There are also studies suggesting a connection between ketonemia in pregnant women and mental children development. The study on 223 pregnant women (35 healthy, 89 with pregestational diabetes, and 99 with gestational diabetes) was published in the *NEJM* in 1991. *β*-Hydroxybutyrate and FFA plasma level as well as urine acetone during pregnancy in mothers and the offspring intelligence quotient aged 2 and again between 3 and 5 yrs old were evaluated. It was found that children of mothers with higher *β*-hydroxybutyrate and FFA concentrations showed a lower level of intelligence. There was no such relationship with acetonuria [36].

In the animal model, keeping the ketogenic diet after birth was associated with a risk of diabetic ketoacidosis, mainly during the first three weeks, and it resulted in a slowdown of further offspring growth [37]. On the other hand, as adults, they were characterized by an increased physical activity and reduced susceptibility to anxiety and depression. It is suggested that the ketone levels interfere with dopamine and serotonin metabolites that are neurotransmitters involved in the processes of anxiety and depression. It cannot be excluded that these anatomical brain changes caused by elevated ketone levels in the fetal life may be a cause of behavioral problems also in later life. A long-term study of ketogenic diet effects suggests possible deleterious effects on memory and other cognitive functions in rats and humans [34].

Carbohydrate disorders are the most common complication of pregnancy nowadays. According to the International Diabetes Federation Atlas from 2017, even 16.2% of pregnant women experience these disorders. The vast majority of 75–90% are gestational diabetes [38]. The data available on the effects of carbohydrate disorders on fetal development provides contradictory information. The first meta-analysis of this problem was published in 2015—12 pieces of research were qualified (none of them was a randomized clinical trial); 9 of which were prospective cohort studies and 2 of them were retrospective studies. Although the authors emphasize the high heterogeneity of the results, it has been proved that the mother's carbohydrate disorders may adversely affect the offspring's development. It was found that children aged 1-2 were characterized with a 41% lower mental development, 31% worse psychomotor development, and even up to 78% lower IQ rates in comparison to their physiological pregnancy counterparts [39].

Despite such observations, Polish, British, or American Diabetes Association does not consider ketone control as a routine recommendation in patients with gestational diabetes.

## 3. Ketone Control in Patients with Diabetes

Methods used to control ketone bodies can be used in self-monitoring. They determine ketones in urine by strip tests or measure *β*-hydroxybutyrate concentration in the capillary blood.

Acetoacetate and acetone concentration is determined in urine by means of so-called legal test that is based on the reaction of the abovementioned compounds with sodium nitroprusside and glycine in alkaline environment. This test does not detect *β*-hydroxybutyrate. This is a semiquantitative sample, which outcome is determined subjectively according to the test strip colour. Urine gives a red colour in conjunction with nitroprusside in alkaline environment. In case of acetoacetic acid or acetone being present in urine, the colour changes into cherry after the acetic acid is added. If ketones are not found in urine after adding the acetic acid, the red colour becomes yellow and green [40]. These measurements, however, do not correspond to the current ketone concentration in the blood; yet, they allow to assess the acetoacetate level from two to four hours prior to the assay [41]. False positive results can occur with the use of drugs containing sulfhydryl groups, for example, captopril, penicillamine, and mesna, whereas high concentrations of ascorbic acid in urine may cause false negative results [42]. In addition, with ketone bodies, we observe an effect similar to the “renal threshold for glucose” phenomenon; in mild ketonemia, only a small amount of ketone bodies is excreted in urine. Accordingly, the preferred method for ketone determination is to assess their level in the blood [10, 43]. There is some scientific evidence that *β*-hydroxybutyrate detection in the blood is a more sensitive and specific method for diagnosing ketonemia [41, 44–46].

This assessment can be performed using the so-called ketometers. The Abbott Precision Xceed Pro® device has been approved by the U.S. Food and Drug Administration (FDA) as a device for measuring *β*-hydroxybutyrate levels at home and hospital. This device can gauge the quantity of *β*-hydroxybutyrate blood concentration between 0 and 8 mmol/l. The test involves applying a blood drop on a single use strip placed in a device. The strip is saturated with *β*-hydroxybutyrate dehydrogenase enzyme. Thanks to this enzyme, *β*-hydroxybutyrate is converted into acetoacetate, which reduces the NAD to NADH at the same time. An electricity flow occurs during this reaction with a current which value is proportional to *β*-hydroxybutyrate blood concentration.

An improperly collected blood sample can affect these results. Hence, the arterial blood, neonatal blood, plasma, and serum should not be used. Moreover, the hematocrit level should range from 30% to 70% to make the measurement accurately. Extra caution should also be maintained in severely dehydrated patients with a severe hypotension, shock, or with hyperosmolar hyperglycemic syndrome [11, 47]. Currently, the Association of British Clinical Diabetologists defines the determination of *β*-hydroxybutyrate blood concentration as the best way to monitor response to diabetic ketoacidosis treatment [48]. Yet, the American Diabetes Association and the Polish Diabetes Association are not in favor of any of these methods [49, 50].

In pregnancy, complicated by diabetes to a significant ketone level increase in comparison with healthy pregnant women has been reported. Current diabetic association guidelines do not recommend ketone routine assessment in pregnant women with diabetes. We believe that the information described in the article justifies starting a discussion on this problem.
